# Regional variations in environmental impacts of NMC 811 production: Comparative LCA study across Visegrad countries

**DOI:** 10.1016/j.isci.2026.115110

**Published:** 2026-02-20

**Authors:** Thaiskang Jamatia, Viera Pechancová, Debashri Paul, Jens Buchgeister, Manuel Baumann, Hüseyin Ersoy, Merve Erakca, Petr Saha

**Affiliations:** 1 Centre of Polymer Systems, University Institute, Tomas Bata University in Zlín, Tr. T. Bati 5678, Zlín 760 01, Czech Republic; 2 Institute for Technology Assessment and Systems Analysis (ITAS), Karlsruhe Institute of Technology (KIT), P.O. Box 3640, Karlsruhe 76021, Germany; 3 University Institute, Tomas Bata University in Zlín, Nad Ovčírnou IV 3685, Zlín 760 01, Czech Republic

**Keywords:** environmental science, energy storage, energy systems

## Abstract

There is a high consensus that decarbonization of the mobility sector is possible by replacing internal combustion engine vehicles with battery electric vehicles. Among the lithium-ion batteries, the NMC 811 battery chemistry is commonly used in electric vehicles. This work evaluates the cradle-to-gate environmental impacts of industrial-scale NMC 811 battery production in the Visegrad countries—Czech Republic, Hungary, Poland, and Slovakia. Norway, with its low-carbon electricity mix, is included for comparison. This study showed how differences in electricity grid profiles influence the environmental impacts of lithium ion battery (LIB) production. The battery production in Poland has the highest global warming potential and Norway, due to its cleaner electricity grid, contributed the least for the same NMC 811 battery production. Hungary, Slovakia, and the Czech Republic reported lower GWP values. These variations highlight the critical importance of decarbonizing electricity grids to reduce emissions associated with LIB production.

## Highlights

• Cradle-to-gate LCA study of NMC 811 battery production in the Visegrad group of countries
• Battery production’s environmental impact depends on a country’s electricity grid profile
• Assessment of GWP for NMC 811 battery production in Visegrad nations and Norway (2019–2024)
• Poland reported the highest GWP (fossil-based grid) and Hungary reported the lowest

## Graphical abstract

**Figure:**
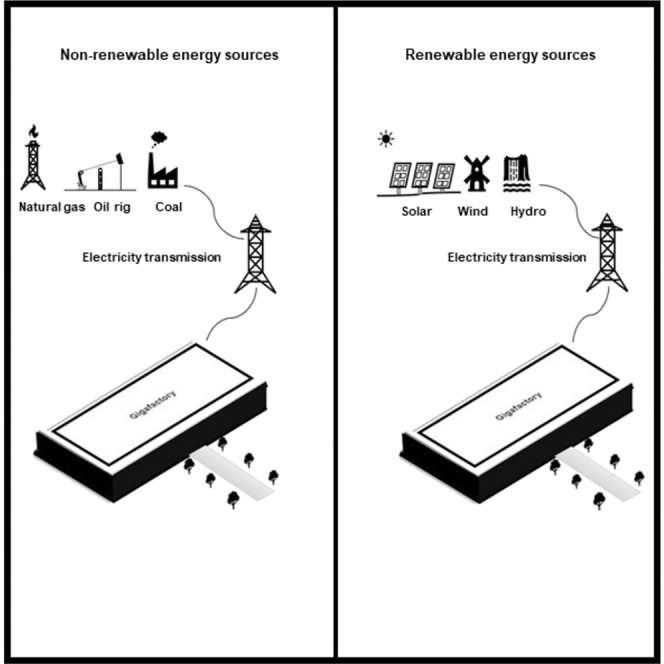

## Introduction

Due to the surge in demand for electric vehicles, the production of Li-ion batteries has seen an exponential growth. To address the pressing issue of climate change, the European Union (EU) set up policies like the SET-Plan, established in 2008 to define, develop, and construct an energy technology policy of Europe.^1^ One of the objectives set was in contributing to the global transition to a low carbon economy by 2050.^2^ This resonates with the EU policy framework to establish battery regulations requiring manufacturers to provide an environmental footprint report before exiting the factory gate.

The usage of batteries, especially lithium ion, is not limited to battery electric vehicle (BEV). Li-ion batteries like LFP (lithium iron phosphate) and, redox flow battery (RFB) have been extensively used in energy storage devices like battery energy storage system (BESS) or hybrid energy storage system (HESS),^3^^,^^4^^,^^5^ hybrid-electric aircraft,^6^ and electric hydrofoil boats.^7^

According to McKinsey’s report,^8^ the transportation industry contributes 28% to the GHG (greenhouse gas) emission in the EU. BEVs are considered eco-friendly compared to the conventional internal-combustion engine (ICE) vehicles, particularly in terms of lower greenhouse gas emissions.^9^^,^^10^^,^^11^^,^^12^ However, there is embedded emission attached to the BEVs which is visible in the production of BEVs, especially associated with batteries. The energy required to manufacture the battery components is high, and majority of the energy demand goes to the production of the cell components, mainly the cathode materials.^13^ In addition, the presence of critical raw materials (CRMs) like lithium, cobalt, manganese, nickel, and graphite also contributes to the environment impacts of battery production. The social implications of cobalt extraction are similarly concerning, especially the mining of the metal in the Democratic Republic of Congo (DRC) where child labor at the mining sites coupled with unfavorable working conditions were reported.^14^

The automotive industry is one of the major sectors that contribute to the economy in the Visegrad countries. The industry is crucial for Europe, as well, as it represents 8% of its GDP.^15^ The number of BEVs purchased by the consumers in 2023 was 14 million, of which 95% of these sales occurred in China, Europe, and the United States together. After China (62%), Europe is the second largest market for the EVs (electric vehicles) representing over 25% of new market sales.^16^ Table 1 shows the vehicle (cars and commercial vehicles) production in the EU in 2023.^17^ Out of almost 14 million vehicles produced in the EU in that year, the Visegrad countries together manufactured 3 million vehicles in 2023. This accounts to 25.90% of the total vehicle production in the EU (International Organization of Motor Vehicle Manufacturers, OICA).^17^ The numbers show the importance of the four-member countries contributing to the automobile industry. To maintain competitiveness, a transition toward electric vehicle production in these countries is inevitable.

**Table 1:** Car production in the EU in 2023^17^

2023 Car production, EU	Total production	Percentage
AT	114,191	0.82
BE	332,103	2.39
CZ	1,404,501	10.09
FI	30,191	0.22
FR	1,505,076	10.81
DE	4,109,371	29.52
HU	507,225	3.64
IT	880,085	6.32
PL	612,882	4.40
PT	318,231	2.29
RO	513,050	3.69
SK	1,080,000	7.76
SL	60,881	0.44
ES	2,451,221	17.61
Sum	13,919,008	

AT, Austria; BE, Belgium; CZ, Czech Republic; FI, Finland; FR, France; DE, Germany; HU, Hungary; IT, Italy; PL, Poland; PT, Portugal; RO, Romania; SK, Slovakia; ES, Spain.

**Figure 1:**
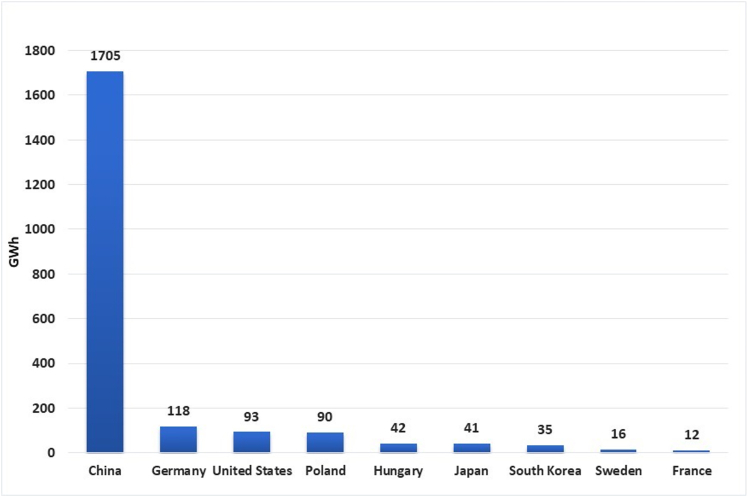
Global top-10 battery manufacturing capacity (GWh) in 2023 (statista.com)

With growing demand for the EVs, the manufacturing of lithium ion batteries (LIBs) is picking up pace across Europe and several Gigafactories are being built and in pipeline.^18^ The leading manufacturer in 2023 in terms of LIB production is China with almost 80% of the total production worldwide (Figure 1). According to another report, Europe produced 185 GWh of LIBs in 2023 and, Poland and Hungary contributed 60% and about 30%, respectively.^19^ In the Visegrad region, LG Chem in Wroclaw, Poland, is the largest Gigafactory with the current annual battery production capacity of 86 GWh.^20^ One of the world’s largest battery manufacturer from China CATL (Contemporary Amperex Technology Co., Limited) is setting up a Gigafactory in Debrecen, Hungary.^21^ Similarly, the Czech government made public for the construction of a new Gigafactory in Karvina^22^ and Gotion-InoBat has signed a Memorandum of Understanding (MoU) with the Slovak government for establishing a new Gigafactory.^23^

Addressing the issues related to the production of batteries for the EVs requires a thorough and comprehensive approach such as life cycle assessment (LCA). It is a standardized method to evaluate the environmental impacts of a product over its entire life cycle. This assessment tool gives an overview and an insight knowledge regarding the sustainability of a product. There are several works conducted and reported in scientific journals on the LCA study of BEVs highlighting key contributing factors on reducing the environmental impacts of BEV productions.^24^^,^^25^^,^^26^^,^^27^^,^^28^^,^^29^^,^^30^ The LCA studies on the environmental impact of LIBs revealed that battery cell production step is one of the most energy-intensive stage.^13^^,^^31^^,^^32^

Burchart-Korol et al.^33^ reported a comparative analysis of the BEVs in Poland and the Czech Republic. The main focus of the study was the electricity generation required to power the charging of the BEVs. The cradle-to-grave assessment evaluated both the current and future production (2015–2050) of the electricity needed to charge the BEVs. Duffner et al.^34^ reported the production of the battery plant location in the 28 EU nations. The authors presented a methodology for assessing and evaluating battery manufacturing plant location choices based on cost of establishing a plant and the scientific and technological knowledge of battery. The work revealed that, based on the scientific and technological knowledge, Germany, France, and the United Kingdom scored the maximum points and the lowest point came from Malta. And, the leaders among the plant location from the cost perspective came from the group of countries geographically located on the eastern side of Europe—Bulgaria, Latvia, and Poland. In another work by Kucukvar et al.,^35^ 27 EU nations were considered to investigate the environmental impacts of the BEVs by considering the electricity mix production of these 27 countries. The study revealed that Finland and the Netherlands contributed the least to the environmental impacts in the production of BEVs since their electricity mix generation has a higher proportion of renewable electricity mix sources (Norway electricity mix was not included in the study). Winjobi et al.^36^ reported on the environmental performance of NMC (nickel manganese cobalt) batteries with varying stoichiometric ratios of the active cathode materials (NMC 111, NMC 532, NMC 622, and NMC 811). The baseline for the study was GREET (Greenhouse gases, Regulated Emissions, and Energy use in Technologies) NMC 111. The work also evaluated on the environmental assessment to understand the supply chain, electricity grid, and production location of the NMC batteries. The regional electricity grids used in the study were the United States, China, Japan, South Korea, and Europe. Coal-based electricity mix generation resulted in higher GHG emissions of the NMC precursors compared to the NMC 111 GREET baseline by 9%, 8%, 3%, and 1% for NMC 111, NMC 532, NMC 622, and NMC 811, respectively. When electricity mix production powered by hydro was studied, a reduction in the GHG emission was observed. Degen et al.^37^ evaluated the energy consumed and the GHG emissions through the LCA study of the Research Factory for Battery Cells (FFB) in Germany. In the study, the authors investigated the energy required and the GHG emissions from each step in the NMC 622 battery production and reported that maximum contribution came from coating and drying, formation, and drying rooms. The work further reports that the GHG emissions of the LIB production are dependent on the location of the manufacturing plant.

The Visegrad group of countries is considered major automotive producers in the EU (25% of the vehicle production in the EU came from this region; Table 1). And, to stay competitive in the automobile industry, there is a need to shift toward EVs. Battery production is an energy-intensive process and the sustainability of EV production depends mostly on the environmental footprints of batteries. As a result, having a good understanding of the impacts of battery production with respect to GWP, energy demand, and emissions related to battery production is important for guiding cleaner industrial strategies in the region. The work by Shafique et al.^38^ reported an LCA study of electric vehicle (EV), plug-in hybrid electric vehicle (PH-EV) and ICE, both petrol and diesel within the geographical setting of Hong Kong. In their study, the authors evaluated the GWP values for the production of an EV that include-li-ion battery, chassis, transmission system, vehicle body, vehicle fluids, generator, powertrain system, traction motor, vehicle assembly, lead acid battery, and electronic controller. The major contributor came from the production of li-ion battery (46%), followed by the production of vehicle body (18%) and vehicle assembly (9%).

### Literature gap, research motivation, and novelty of the work

There are a few LCA studies conducted on the production of LIBs in the Czech Republic, Hungary, Poland, and Slovakia.^33^^,^^37^^,^^39^^,^^40^ The article by Korol et al.^33^ reported about the charging of EVs in the Czech Republic and Poland and its environmental impacts of the energy used during charging. Degen et al.^37^ focused on the battery production in Europe, and the results demonstrated that location of a battery production plant has a significant influence on the GWP values during cell production. However, no such work has been reported in the literature that collectively investigates the battery production for EVs in these Visegrad countries together provides a comparative analysis of their respective outcomes.

## Results and discussion

### GWP100 values of battery pack production for different electricity mix

The LCA study in this work examines the NMC 811 battery production at different geographical locations with varying electricity grid. The GWP100 (the average warming potential over 100 years) associated with the battery production is an important indicator that reflects the environmental impact of GWP (GWP will be used throughout the text implying GWP100) emissions from the manufacturing process. The GWP value of LIB production varies significantly based on the battery chemistry, methods of production, and regional or geographical locations. Among various battery chemistries, NMC has many variants like NMC 111, NMC 333, NMC 622, and NMC 811. The variants with higher nickel content and low cobalt content tend to have low GWP on the lower end of the spectrum and NMC 811 has the least of all, comparatively.^13^^,^^36^

**Figure 2:**
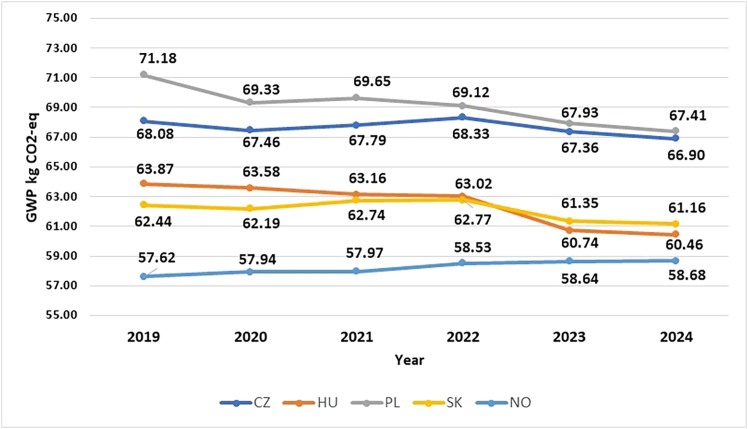
GWP values (per kWh) for NMC811 battery production in CZ, HU, PL, SK, and NO from 2019 to 2024

**Figure 3:**
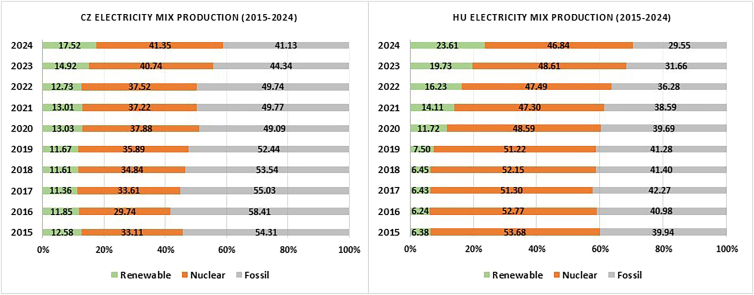
Electricity production, excluding import of electricity, in the Czech Republic and Hungary in 2015–2024 (energy-charts)

**Figure 4:**
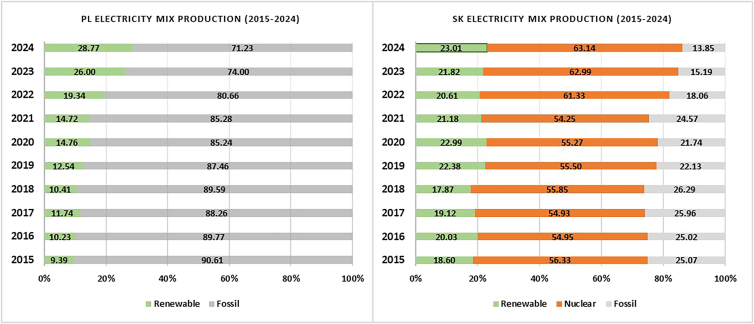
Electricity production, excluding import of electricity, in Poland and Slovakia in 2015–2024 (energy-charts)

The line graph in Figure 2 illustrates the GWP values of the battery production in the Visegrad countries and Norway over a period of 6 years, 2019–2024. When the GWP values for battery production for each country are examined collectively, a connection can be drawn between the observed patterns in emissions with underlying changes in the electricity generation mix. The gradual integration or expansion of the renewable energy shares and the reduction of fossil fuels influence can be observed in Figure 2. Further insight into this relationship can be obtained by considering the electricity production profile of the repspective countries. Table 4 depicts the electricity production profile for 2024 in the Visegrad groups and Norway, while Figures 3 and 4 illustrates the bar chart of electricity generation from 2019–2024 clubbed together to three sources- renewable, nuclear and, fossil. Referring to Figure 3, the contribution to the electricity grid of the Czech Republic is around 52.40%, 35.89%, and 11.67% for fossil, nuclear, and renewable, respectively, in 2019. Over the years until 2024, the reliance on fossil reduced to around 41.35% and not much of a change or development was observed in nuclear and renewable sources. This is reflected in the GWP values for the manufacturing of battery in this time period too, though with slight changes from 68.08 kg CO2-eq/kWh in 2019 to 66.90 kg CO2-eq/kWh in 2024.

On the other hand, Hungary updated their grid profile by increasing their renewable share from 7.5% to 23.61% and simultaneously reduced their fossil share from 41.28% to 29.55% from 2019 to 2024 (Figure 3). This is also reflected in the GWP values of battery production where a drop from 63.87 to 60.46 CO2-eq/kWh could be seen (Figure 2). The contribution in the renewable sector is from the installation of the solar panels in Hungary; it rose from a mere 0.6% in 2019 to 17.6% in 2024. Furthermore, hydropower makes up roughly about 1.96% to the grid mix. These factors explain why in 2023 and 2024, Hungary’s GWP values are lower than Slovakia’s despite fossil fuel contributing about 30% of the electricity production in Hungary and c.a. 15% in Slovakia (Figures 3 and 4).

Among the four Visegrad countries, Poland has the highest share of fossil-based grid profile, with 74% contribution (Figure 4). As can be seen in Figure 4, and in the previous GWP graph of cell production in Poland (Figure 2), it contributes the most when compared to the rest of the national grid in this study. However, when referring to Figure 4, the national grid of Poland reduced their dependence on fossil from 90.61% to 71.23%. This is a significant reduction in the usage of fossil fuels for electricity generation. The country is heavily reliant on fossil hard coal. Another notable point to be mentioned is that Poland has the highest share of renewable sources for electricity production among the Visegrad countries in 2024. Standing at 28.77%, like Hungary, the nation is investing on wind onshore, which contributes about 15% from the total share of renewable sources. It is also to be noted that 2020 onwards, the country started investing on the installation of solar panels. The percentage share rose from 1.26% in 2020 to 11.03% in 2024. These numbers resonate with the GWP values of battery production in Poland from 2019 to 2023 (Figure 2). It can be seen that for countries with electricity mix generated from fossil fuels results in higher GWP values (67.41 kg CO2-eq/kWh), as in Poland. On the other hand, Norway with 99% of its electricity production from a renewable source has the least GWP values (58.68 kg CO2-eq/kWh). The GWP values of the battery production for the electricity mix modeled from 2024 is depicted in Figure 2.

The timeline from 2015 to 2024 shows that Slovakia increased their electricity production of nuclear source from 14.54 to 18.19 TWh. This is the only country in the Visegrad countries with the least contribution from fossil-based source, 13.85% in 2024. In Slovakia, of the 13.85% fossil, 2.79% is brown coal, 0.51% is hard coal, and 7.06% is fossil gas. For Hungary, of the 29.5% fossil, about 7.28% is brown coal (lignite) and 0.79% is fossil hard coal and 19.82% is natural gas. For Slovakia, the operation of conventional power plants and combined cycle power plants (CCPPs), for electricity production from natural gas, contributes 1.4% and 2.1%, respectively. In the case of Hungary, the percentage contribution from CCPs is 13% and conventional power plant contributes 1.1%. Comparing the energy efficiency of both the conventional power plant and the CCPPs for the production of electricity from natural gas, the CCPPs have efficiency of about 55% and the conventional power plant efficiency is around 33%.^41^ According to the work reported by Viola et al., the electricity production from natural gas, using the CCP contributes relatively less to GWP as compared to electricity production using the conventional power plant.^42^

In the renewable sector, 14.54% of their share is from hydro run-of-river. Referring to Figure 2, the results of the GWP values from 2019 to 2024 can be corelated with electricity production from Figure 4. The GWP values of these countries in the EU are comparable and falls within the range of the published articles in the literature.^36^^,^^43^ Among the Visegrad group of countries, Slovakia has the most percentage of electricity produced from nuclear, 63.14% in 2024, followed by 23.01% from renewable sources. Slovakia is one of the major automakers in Europe with its annual vehicles manufactured crossing over a million and exporting to several nations.^44^ In 2022, Slovakia topped the chart in the production of car manufactured per capita with 184 cars produced per 1,000 inhabitants followed by the Czech Republic (115 cars per 1,000 residents).^45^

### Contribution of the electricity consumption in battery production

**Figure 5:**
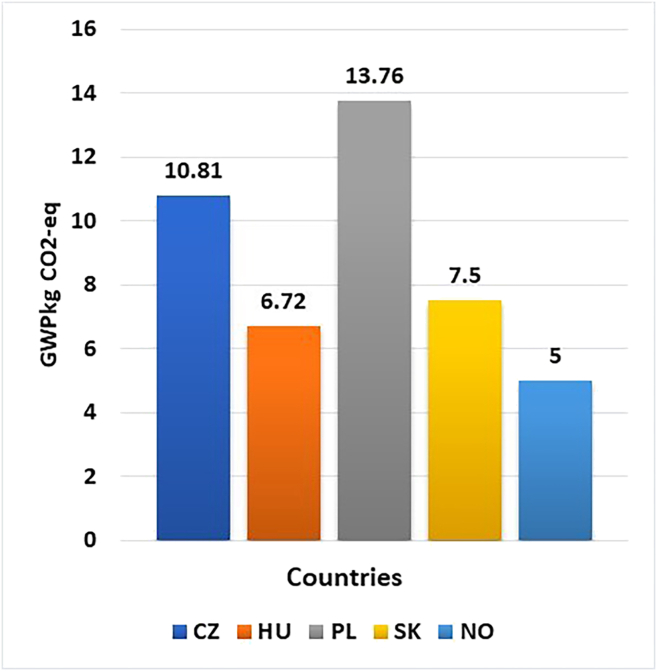
GWP of energy demand per kWh (electricity and heat) during battery production, 2024 electricity mix

**Figure 6:**
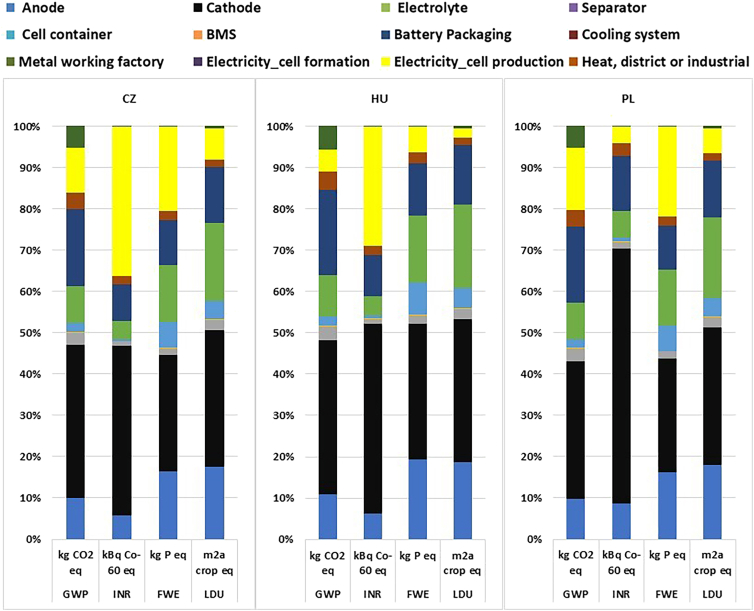
Impacts of 1 kWh of NMC 811 battery production on GWP, INR, FWE, and LDU in CZ, HU, and PL, 2024 electricity mix

**Figure 7:**
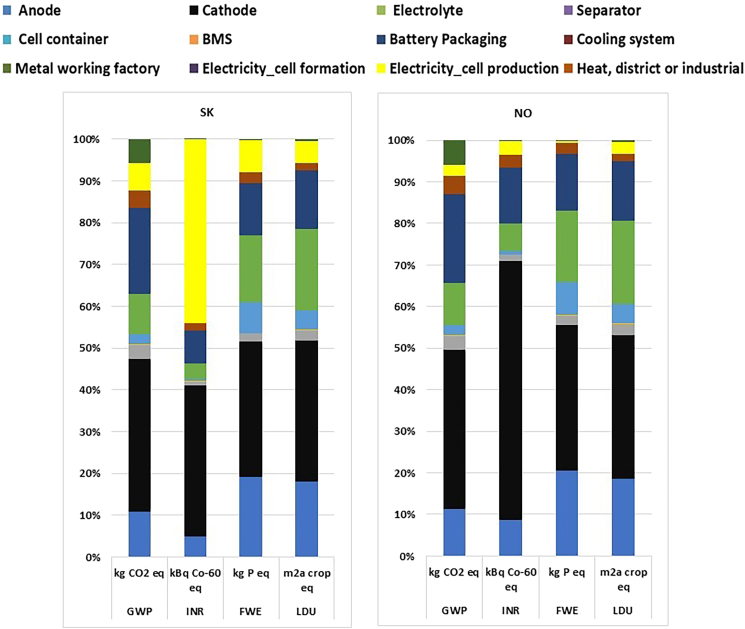
Impacts of 1 kWh of NMC 811 battery production on GWP, INR, FWE, and LDU in SK and NO, 2024 electricity mix

In the study, the energy demand for the battery production is sourced from electricity and heat (from natural gas). The energy demand in the manufacturing of 1 kWh of NMC 811 battery is depicted in Figure 5. In this work, the GWP values for the battery production were taken for the electricity and natural gas. The energy demand values are procured from a work by Kuki et al. where the energy used was calculated from a CATL factory in Debrecen, Hungary.^39^ The projected annual use of electricity and natural gas in the factory (including the factory offices) was 90,000,000 m^3^ and 533 GWh, respectively. As can be seen in Figure 5, the highest energy demand contribution to the GWP value is from Poland. In Poland, to manufacture 1 kWh of battery, the energy consumption is 13.76 kg CO2-eq/kWh of GWP (Figure 5). It constitutes 20.56% of the energy consumption from electricity and heat production from natural gas contributed 0.002%. The manufacturing of the NMC 811 battery in the remaining country grid has 16.16%, 11.20%, and 10.04% contribution for the Czech Republic, Slovakia, and Hungary, respectively (Figures 6 and 7). As for Norway, about 7.45% is the percentage contribution to the total GWP during battery production (Figure 7). Among other factors, the reduction in the carbon intensity is proportional to the GWP from the battery production. Poland is the largest producer of LIB among the Visegrad group in Europe.

**Figure 8:**
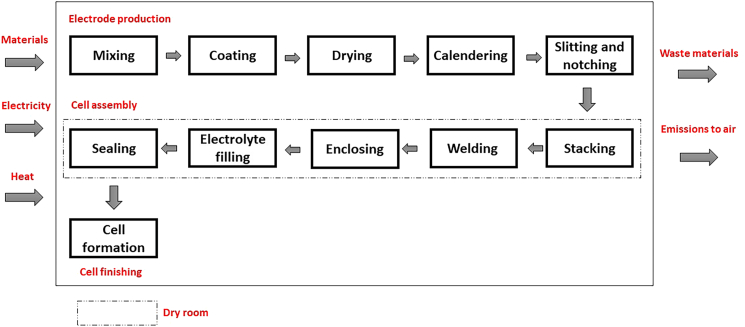
Process flow diagram of cell production

The GWP values for the production of NMC 811 battery for the year 2024 is shown in Figures 6 and 7. An overview of the main stages in cell manufacturing is illustrated in the process flow diagram in Figure 8. Within this production sequence, and across all the cases shown in Figure 2, the production of cathode is mainly responsible for the GHG emissions and causes major environmental effects,^46^^,^^47^^,^^48^ as is the case in this study as well (Figures 6 and 7). The electrode production (mainly from coating/drying of anode and cathode) and the use of dry room are the most energy-intensive steps in the manufacturing of a cell.^49^^,^^50^ The other factors that heavily contribute are mining and extraction of the upstream raw materials like lithium, nickel, cobalt, and aluminum.^51^^,^^52^^,^^53^

The energy consumption (electricity demand) for producing 1 kWh of battery reported in this study (sourced from Kuki et al.) is 34.6 kWh. For a 40 GWh of annual production, the total electricity demand for annual production amounts to 1.384 million kWh (electricity demand annually = factory annual capacity ∗ energy demand per kWh of battery production). This translates to 1.384 TWh of the energy demand to produce 40 GWh of battery production annually. The electric production in each country in 2024—Czech Republic, Hungary, Poland, and Slovakia, produced is 77.11 TWh, 60.97 TWh, 171.24 TWh, and 36.80 TWh, respectively. Table 2 below summarizes the electricity production in 2024, energy demand for battery production, and the share of from each country.

**Table 2:** Share of national electricity demand for setting up a 40 GWh Gigafactory in the Visegrad group of countries

Country	Electricity production (2024) (TWh)	Annual electricity demand (TWh)	Percentage share of national electricity demand
Czech Republic	77.11	1.384	1.79
Hungary	60.97	1.384	2.27
Poland	171.24	1.384	0.81
Slovakia	36.80	1.384	3.76

From the table, it can be seen that, depending on the country’s electricity grid mix, the electricity demand ranges from 0.81% to 3.76%. This can be a noticeable addition to the national grid load, especially for a country like Slovakia and Hungary.

Ionizing radiation relates to damage to human health and the ecosystems linked to the emissions of radionuclides during a number of human activities. Mining and processing of raw materials, treatment and handling of radioactive materials, and burning of coal indirectly generate ionizing radiation by releasing naturally occurring radioactive materials (NORM) into the environment. Figures 6 and 7 show the contribution to the ionizing radiation (INR) during the NMC 811 battery production. Slovakia (43.89%), Hungary (28.71%), and the Czech Republic (36.1%) have the maximum percentage share of electricity generation from nuclear energy (Figures 3 and 4). The numbers are clearly reflected in the contribution of IR during the battery production. These three countries contribute 50% or more from electricity production to the ionizing radiation. More environmental impacts especially from ionizing radiation occurs if a battery manufacturing plant with a significant share contributing from nuclear power generation. This is attributed to the mining and extraction of, mainly, uranium, their processing and waste management. On the contrary, Poland and Norway do not operate nuclear power plants contributing 3.95% and 3.23%, respectively, of ionizing radiation during the manufacturing of the NMC 811 battery production. The emission, however, can be associated indirectly from their processes of electricity generation rather than the primary energy generation process.^54^ Coal-fired plant may contribute to IR as coal contains natural radio nuclides like uranium, thorium, and radium.^55^ Coal ash containing these radionuclides is emitted into the environment when burned in power plants.^56^

Freshwater eutrophication is the presence of nitrogen and phosphorus in water body (non-saline water) results in the algal growth, hypoxia, and depletion of marine ecosystems. Raw material extraction and its processing (especially lithium, nickel, manganese, and cobalt mining), and battery production are one of the key contributors to freshwater eutrophication. According to Accardo et al., nickel and cobalt extraction accounted for 56% and 20% to the impact category, respectively.^57^ In the manufacturing stage, the production of cathode is the main contributor to the impacts caused to the freshwater bodies followed by the production of anode. The energy consumption is high in the electrode production as it requires high-temperature processing which significantly increases the energy and electricity demand. Norway with 99% renewable electricity grid contributes the least to freshwater eutrophication and, Hungary and Slovakia contributed around 15%. The percentage share of fossil in their national grid is 6.12% and 9.50%, respectively. Conversely, the electricity grid from Poland and the Czech Republic contributes about 22.41% and 21.05%, respectively, to the battery production. The waste from the coal-fired thermal powerplant released into the water contains toxic chemicals in the form of phosphates, heavy metals, and nitrogen compounds. These toxic chemicals aid in the algal growth leading to eutrophication. In addition, according to Dones et al., 84% of the freshwater eutrophication comes from mining due to the release of phosphates in the water bodies.^58^ Gaete-Morales et al. also highlighted on the role a coal-based electricity mix play in significantly contributing to freshwater eutrophication.^59^

In this work, the majority of the contributions in land use are from the manufacturing of the cell components—anode, cathode, and electrolyte (together they contributed around 80% to LDU), mainly from mining and processing of the electrode materials (Figures 6 and 7). And when it comes to electricity production, there are variances in land use impacts. For fossil-based, land is primarily occupied or used for coal mining, drilling land for natural gas, and for renewable source, wind, and solar farms can be installed on agricultural land. Electricity generation from coal and its extraction, especially hard coal, has several environmental impacts, other than greenhouse gas emissions, especially in land use. According to a report by the United Nations Economic Commission for Europe (UNECE),^60^ the extraction of coal contributes the most in the generation of electricity from coal. The unit used in the study is points (quality of soil occupied, a unit related to the quality of a land). For land use, the coal-powered plant affects the land both directly and indirectly. The direct land transformation includes mining of a land, damage to the soil and ecosystem in the mining area due to the extraction of coal. The indirect transformation includes upstream processes like building and operation of the coal mines and its infrastructure. Having largely dependent on coal for electricity generation, this is reflected in the land use contribution in Poland and the Czech Republic (Figure 6).

As illustrated in the tree diagram in Figure S2, the preparation of cathode active material involves the synthesis of NMC hydroxide precursor followed by calcination where oxide active material is the product formed. The cathode active materials production in Figure S2 is from the Czech Republic, and the electricity mix data modeled are for the year 2024. Cathode production is one of the most energy intensive processes. Also, similar trends are expected for Hungary, Poland, and Slovakia. In the cathode production, the active material for the preparation of oxide material of NMC and prior to that, the preparation of hydroxide of NMC is an energy-consuming process. Dai et al.^48^ reported on the NMC 111 powder production process. In their work, the industrial production of the active cathode materials was reported where it was analyzed that co-precipitation step followed by calcination process were the most energy-intensive process. It was reported that to produce 1 kg of hydroxide of the NMC active materials via co-precipitation process, 42.6 MJ energy was consumed. The calcination step consumed 25.2 MJ. This finding aligns with Figure S2 in this work, where these two processes exhibit the highest energy contributions.

Hydropower significantly affects land occupation due to reservoirs. Among the renewables, hydropower (depending on the size of reservoirs as higher reservoirs occupy more land) contributes the most and wind the lowest to land use. The land occupation in hydropower includes both agricultural and urban land transformation. It is dependent on the geography and topography of a land. In this study, Norway accounts for most of the land-use impacts linked to electricity generation, primarily due to its reliance on hydropower. From the 99% of renewable share in the electricity mix for 2024, Norway generates about 89% from hydropower alone. The direct land transformation associated with the installation of onshore wind mill on an agriculture land is relatively minimum.^58^

Electricity generation from nuclear power includes mining, milling, conversion, enrichment, fabrication, power plant, and fuel disposal.^61^ According to Fthenakis et al., the building of the infrastructure, power plant contributed the most to land use (m^2^/GWh). However, Fritsche et al. reported that the direct land occupation required to establish nuclear power facilities is relatively small. Czech Republic, Hungary, and Slovakia have high percentage of electricity share mix from nuclear power.^62^ The contribution from the nuclear source is reflected on the LDU impact category in the NMC 811 battery production (Figures 6 and 7). In addition, to have an international benchmark, the GWP values of the current study are compared with China. The GWP value for the production of the NMC 811 battery in China is 72.48, (Figure S1). The data for electricity market mix was sourced from the ecoinvent database. The documented database was last updated in December 2023. It is, therefore, assumed that the electricity data are from 2022/23. Comparing the GWP value of battery production from China with the rest of the Visegrad group of countries from 2022/23, China is the highest contributor. This results from the fact that about 61.20% of the electricity is powered by coal, 3.08% from natural gas, and 0.814% from oil, amounting up to 65.09% from fossil-based for electricity generation.^63^ In terms of proximity to the electricity production of China, Poland’s electricity profile is the closest among the Visegrad countries (Figure 5). When compared to the work by Dai et al., they reported a GWP value of 72.90 kg CO2-eq. Although the battery chemistry is NMC 111, this is the closest reported work in the literature at the pack level for Chinese production.

### Comparative analysis of the current work with the reported works

To systematically compare several LCA works on LIB production with the current work, these are the parameters taken as benchmarks-functional unit, battery chemistry, system boundary, cumulative energy demand, GWP, and geographical region (Table 3). The table consistently compares the quantitative matrices and interprets differences based on battery chemistry and on regional context. Kim et al.^64^ conducted an LCA study of Ford Focus electric vehicle. The inventory for the bill of materials (BOMs) was collected from the manufacturer. Their study reported the GWP values of 140 kg CO2-eq/kWh at the pack level and 89 kg CO2-eq/kWh at the cell level. In another work, Dai et al.^48^ visited a couple of leading battery manufacturers in China and procured the BOM of the NMC 111 battery pack. The annual production reported being 2 GWh of battery cells. The combined energy demand during the manufacturing process was measured to be 47.20 kWh/kWh_cell_, and the GWP values at the pack level was calculated as 72.90 kg CO2-eq/kWh. Chordia et al.^65^ conducted an LCA study of a 16 GWh battery production from Northvolt in Sweden. The authors considered two locations for the Giagfactories, Sweden, and South Korea. The electricity grid of Sweden is favored more toward renewable sources and the South Korean electricity mix is mostly fossil-based. In line with this, the GWP values for cell production are 50.40 and 109 kg CO2-eq/kWh for Sweden and South Korea, respectively. Sun et al.^13^ reported a 30 GWh annual battery production from another LIB manufacturer in China. The NMC 622 battery cell with 1.18 kWh/kg specific energy reported a combined energy demand of 29.60 kWh/kWh_cell_. The GWP value of the battery chemistry from the large-scale production plant was 93.5 kWh/kWh_cell_. Kuki et al. reported on three leading Gigafactories located in Hungary—CATL, Samsung, and SK Innovation. The annual production capacities for these Gigafactories are 40, 26, and 30 GWh, respectively. The battery chemistry is NMC 811 for both CATL and SK and NMC for Samsung. In their gate-to-gate study, the GHG per kWh of battery reported was 28.11, 10.95, and 9.91 kg CO2-eq for CATL, Samsung, and SK, respectively. For all the Gigafactories, the energy consumption data were collected from publicly available data sources. The energy consumption values were calculated accordingly and are shown in Table 3.

**Table 3:** Reported works on large scale battery productions at various geographical locations

Battery chemistry	Cell geometry	Cell-specific energy (kWh/kg)	Annual cell production (GWh)	Cumulative energy demand (kWh/kWh_cell_)	GWP (kg CO_2_-eq/kWh_cell_)	System boundary	Factory location	Source
LMO/NCM	pouch	0.08	–	33.33 kWh/kg_cell_	89.0	CTG	S. Korea	Kim et al.^64^
NMC 111	prismatic	0.19	2	47.20	72.90 (pack)	CTG	–	Dai et al.^48^
NMC 622	cylindrical	–	7 (theoretical output)	41.48	10.33	GTG	DE	Degen et al.^37^
NMC 622	cylindrical	–	7 (theoretical output)	41.48	4.54	GTG	SE	Degen et al.^37^
NMC 622	cylindrical	–	7 (theoretical output)	41.48	5.38	GTG	FR	Degen et al.^37^
NMC 622	cylindrical	–	7 (theoretical output)	41.48	8.66	GTG	HU	Degen et al.^37^
NMC 622	cylindrical	–	7 (theoretical output)	41.48	18.83	GTG	PL	Degen et al.^37^
NMC 622	cylindrical	–	7 (theoretical output)	41.48	9.68	GTG	UK	Degen et al.^37^
NMC 811	cylindrical	0.21–0.24	16	no. of cells not mentioned	50.40	CTG	SE	Chordia et al.^65^
NMC 811	cylindrical	0.21–0.24	16	no. of cells not mentioned	109.0	CTG	S. Korea	Chordia et al.^65^
NMC 622	–	0.18	30	29.60	93.5	CTG	CN	Sun et al. (2022)^13^
NMC 811	–	–	40	34.6 (pack)	10.95 (pack)	GTG	HU	Kuki et al. (2025)^39^
NMC and NCA	–	–	26	28.6 (pack)	NA	GTG	HU	Kuki et al. (2025)^39^
NMC 811	–	–	30	29.4 (pack)	9.91 (pack)	GTG	HU	Kuki et al. (2025)^39^
NMC 811	prismatic	0.338	40	34.6 (pack)	57.62–71.18 (pack)	CTG	CZ, HU, PL, SK, NO	Current work

LMO, lithium manganese oxide; NMC, sodium nickel cobalt; CTG, cradle-to-gate; GTG, gate-to-gate; S. Korea, South Korea; NA, not available; DE, Germany; SE, Sweden; CN, China; HU, Hungary; CZ, Czech Republic; PL, Poland; NO, Norway.

In summary, this work reported the NMC 811 battery production of a 40 GWh annual production across the Visegrad countries and, Norway is used as a benchmark for best practises as it generates about 99% of the electricity from a renewable energy source. The objective of the study is to encourage on the production of battery production in the EU, especially in these countries and the commitment to phase out electricity generation from non-renewable sources like coal or a fossil-based energy source.

Through the LCA study, the GWP values of the NMC 811 battery pack production in 2024 varied from 60.46, 61.16, 66.90, and 67.41 kg CO2-eq/kWh for Hungary, Slovakia, Czech Republic, and Poland, respectively. The fact that Norway has electricity grid generated mostly from the renewable sources, with 99% of the grid mix generated from hydro and wind energy, it is the best-case scenario. Norway reported 58.68 kg CO2-eq/kWh of GWP for battery pack production. The study shows the potential of improvements in reduction of greenhouse gas emissions using renewable electricity production mix. The result sheds light on the environmental trade-offs associated with electricity production from nuclear energy. Although nuclear energy contributes to lower a GWP, countries like Slovakia, Hungary, and the Czech Republic, heavily relying on nuclear energy for electricity generation, its impacts were observed due to the radiation associated with nuclear energy. By focusing on the regional grid mixes, it could be seen that the fossil-reliant Poland is investing on the installation of solar panels (the percentage share was 1.26 in 2020 and 11.03 in 2024). Moreover, apart from investing in the installation of solar panels, Poland has also reduced the reliance on coal in the last 10 years TWh (90.61%–74% from 2015 to 2024). This is reflected in the GWP values on the battery production in the last 6 years from 2019 to 2024.

Focusing the transition to renewable electricity grid will be help the EU strive for carbon neutrality. Wind energy set to play a crucial in adapting renewable-based electrification. The findings indicate that energy mix is one of the critical drivers for the reduction of the environmental impacts change being sensitive to electricity grid composition.

### Limitations of the study

As mentioned earlier, the data of the total energy demand were procured from the work by Kuki et al.^39^ In their study, the authors collected data from a leading battery manufacturing company, CATL, located in Debrecen, Hungary. The CATL Gigafactory is expected to begin production in 2025 with an annual capacity of 40 GWh. Though the battery chemistry studied was NMC 811, the battery and cell specifications were not disclosed. These are important factors as the battery capacity and power have an influence in the overall GWP emission.

Further, the inventory for the NMC 811 battery at the cell level was taken from BatPac^66^ and Crenna et al.^67^ and the non-cell components like BMS, packaging, and cooling system cell were taken from Ellingsen et al.^68^ The use of secondary data in this study creates uncertainty which could be substantial and could alter the LCA results depending on the quality of data collected. In addition, the battery materials were not individually assessed in this work as the focus was more on the contribution of electricity mix, at different geographical locations, to the manufacturing of the battery.

In the work, attributional LCA was used to study calculate the environmental impacts of NMC 811 battery production using average electricity mix data from 2019 to 2024. However, the dynamics of electricity generation due to the demand caused by the establishment of gigafactories is beyond the scope of the study. Future work could be directed in the assessment of consequential LCA for the evaluation of potential future grid changes.

In this work, the cumulative energy demand associated with the production of battery is sourced from Kuki et al., who obtained it from CATL Gigafactory in Debrecen, Hungary. The Gigafactory is yet to run in its full capacity. As a result, the authors calculated the projected average annual electricity and natural gas consumption, encompassing all the major battery production processes, which include energy consumption related to dry room. Given that Kuki et al. did not specify individual process data (it was a top-down approach), we interpreted the reported NMP recovery and dry room recovery as inclusive of all the processes and operations. Although this study did not conduct an uncertainty analysis, effort was taken to ensure consistency among datasets and the cumulative energy demand value was assessed in light of its applicability to the system boundary of this study.

## Resource availability

### Lead contact

The delivery of further information and resources will be fulfilled by the lead contact, Thaiskang Jamatia (jamatia@utb.cz).

### Materials availability

This study did not generate new unique reagents.

### Data and code availability

• The data referenced in this article will be shared by the lead contact upon request.
• This paper does not report original code.
• Any additional information required to reanalyze the data reported in this paper is available from the lead contact upon request.

## Acknowledgments

This work was supported by the Horizon Europe project TwinVECTOR (grant agreement no. 101078935) and by the Horizon Europe project SOLiD (grant agreement no.: 101069505) funded by the European Union.

## Author contributions

T.J., conceptualization, data curation, investigation, methodology, validation, visualization, writing – original draft, and writing – review and editing; V.P., conceptualization, formal analysis, funding acquisition, project administration, resources, supervision, writing – original draft, writing – review and editing; D.P., conceptualization, data curation, investigation, writing – original draft, and writing – review and editing; J.B., investigation, validation, visualization, writing – original draft, and writing – review and editing; M.B., investigation, validation, visualization, writing – original draft, and writing – review and editing; H.E., investigation, validation, visualization, writing – original draft, and writing – review and editing; M.E., investigation, validation, visualization, writing – original draft, and writing – review and editing; P.S., funding acquisition, resources, supervision, validation, writing – original draft, and writing – review and editing.

## Declaration of interests

The authors declare no competing interest.

## STAR★Methods

### Key resources table

**Table:** 

REAGENT or RESOURCE	SOURCE	IDENTIFIER
**Deposited data**		

Background data in this LCA study	ecoinvent (v3.10)^69^	https://ecoinvent.org/database/
Inventory: battery pack and cell	BatPac (v5.0)^66^, Crenna et al.^67^ Ellingsen et al.^68^ and Dai et al.^48^	https://doi.org/10.2172/1877590, https://doi.org/10.1016/j.resconrec.2021.105619, https://doi.org/10.1111/jiec.12072 and https://doi.org/10.3390/batteries5020048
Energy consumption value	Kuki et al.^39^	https://doi.org/10.3390/environments12010024

**Software and algorithms**		

SimaPro	SimaPro (v10.1.0.2)	https://account.simapro.com/s/login/?ec=302&startURL=%2Fs%2F

### Experimental model and study of participant details

Omitted, as our study does not involve biological models.

### Method details

The LCA is conducted using SimaPro (v10.1.0.2) and the methodology is standardised according to ISO 14040 and 14044 and consists of the following phases.

#### Functional unit

1 kWh of the nominal capacity of the battery pack is the defined functional unit.

#### Goal and scope definition

The LCA study includes the environmental evaluation and assessment of NMC 811 battery. The study compares the environmental impacts of the NMC 811 battery production at various geographical locations in the Visegrad countries and Norway.

The goal of the study is to evaluate the NMC 811 battery production taking into consideration the different geographical locations (Czech Republic, Hungary, Poland, and Slovakia) with varied electricity mix generation. Norway electricity mix (99% renewable electricity mix production) is chosen for comparison purpose. In this study, the major contributing causes are factored in for the carbon footprint in the form of global warming potential (GWP) for a duration of 100 years. The GWP value during battery production vary on its geographical location. The GHG emission levels during battery production vary on its geographical location. The variation is subject to the source of electricity generation that ranges from fossil-based energy like coal, natural gas oil and renewable energy like solar, wind and hydropower. The outcome of the study would indicate to what extent would the geographic location (source of electricity mix production) of a battery manufacturing site have an effect on the GWP value of the NMC 811 battery.

#### System boundaries

The study includes the cradle-to-gate system boundary the production of the battery (including battery management system (BMS), packaging and coolant system) before it leaves the gate of the factory. The cathode battery chemistry investigated in this study is the NMC 811.

#### LCI (life cycle inventory)

The LCI is one of the processes in the LCA study. This step quantifies the inputs like raw materials, energy supplied, and outputs like waste generated, emissions to air, of a product throughout its life cycle stages. In this step, the inventory data of the NMC 811 battery is compiled according to the ecoinvent (v3.10) guidelines. Most primary data for building inventories of the published studies related to LCA were procured in cooperation with battery manufacturing firms.^13^^,^^64^^,^^65^ Consequently, company confidentiality policies limit the level of detail that could be disclosed in the published study. The secondary data employed can be a combination of data collection from scientific literatures or publicly accessible information available on the company’s website. In this work, the data for all the cell components were sourced from BatPaC v5.0, Argonne National Laboratory (ANL),^66^ Crenna et al.^67^ and the non-cell components like BMS, Coolant system and Packaging were procured from Ellingsen et al.^68^ and Dai et al.^48^ The compilation of the detailed inventory from the sources are presented in the supplemental information (Tables S1A–S1E). The selection of the sources for building the bill of materials (BOM) for this work are based on the transparency and completeness of the inventories provided by the authors. BatPaC, in particular, offers numerous options to select a battery chemistry from its list and provides the flexibility to design a battery pack based on energy density, power requirements and other relevant factors.

The LCI basically detects and quantifies the environmental inputs and outputs linked to a product over the course of its complete life cycle. This is considered an important step of the LCA method as the quality of the LCI determines the quality of the life cycle impact assessment result.

The LCI is typically a tedious task that basically includes data collection and modelling of a subject (or a product). Moreover, another important aspect to look for while collecting inventories is that the product system has to be in line with the goal and scope definition.

#### LCIA (life cycle impact assessment)

The impact assessment methodology used during the LCA study is ReCiPE 2016 Midpoint (H) midpoint. The GHG emissions and other midpoint impact categories like ionising radiation (INR), freshwater eutrophication (FWE) and land use (LDU), the energy required for manufacturing LIBs is calculated for different production location in Europe.

The production in the regional variations in the battery powered electric vehicles remains to be understood. Therefore, understanding the LIB manufacturing and its effects on the environment is therefore crucial to sustainable battery production and its development.

#### Interpretation

In the final phase of LCA study, the results are interpreted in accordance with the stated goal and scope. The conclusion from an LCA study should be in line with the ISO standard. During this phase, the findings from LCI and LCIA are quantified and evaluated. The important thing about the step is in assessing the completeness and consistency of the results obtained and understanding how robust the LCA results are under different scenarios (uncertainty and sensitivity analyses).

#### Battery specification

The mass of the battery at the pack level is 371 kg. The cell mass is 222 kg. It is a prismatic cell with the configurations of 300 cells per pack, 30 cells per module and 10 modules per pack. It is a 75-kWh battery and the energy density of the cell is 337.97 Wh/kg which is similar to the work reported by Kallistas et al. for the NCA (Lithium Nickel-Cobalt-Aluminium Oxide) battery chemistry.^40^ More information is provided in the supplementary file (Table S1A). The NMC 811 cell chemistry has a graphitic anode, synthetic graphite. The positive current collector used is aluminum foils and the negative current collector used is copper foils. More information on the battery parameters can be found in Table S1B.

#### Battery pack

The battery pack is a collection of the cells, BMS, packaging and cooling system that forms an enclosure providing power to an electric vehicle (Tables S1B–S1E).

#### Cell components

##### Battery cell

The most important component of a battery pack is the battery cell. The battery cells chemically store energy that powers a BEV. The NMC 811 cell consist of two electrodes- anode and cathode, separated by an electrolyte and a separator. The cell components are housed in a cell container. Synthetic graphite is the anode and the cathode active material.

##### Anode

The anode is the negative electrode comprising of the anode paste and the negative current collector (copper). The anode paste is composed of synthetic graphite, carbon black (for better conductivity) and carboxyl methyl cellulose (CMC) and styrene butadiene (SB) are the binders.

##### Cathode

The cathode paste consists of the oxides of nickel, manganese and cobalt in 8:1:1 ratio. Additionally, just as in the anode paste, carbon black is added to enhance the conductivity and the polymer polyvinylidene difluoride (PVDF) function as a binder. NMP is used as a solvent in the preparation of cathode paste. The solvent is recovered and it is one of the most energy-inefficient processes. The NMP recovery data is procured from the work by Crenna et al. where 98% of the solvent is recovered and reused. Since, the quantitative analysis of the high-emission stages, like NMP recovery and the energy demand from dry room, was procured from literature sources, the authors acknowledge that the data was based on assumptions backed with sources from already published work.

##### Electrolyte

Lithium hexafluorophosphate (LiPF_6_) was the electrolyte used and the solvent used is ethylene carbonate (EC) and dimethyl carbonate (DMC) in a 1:1 ratio. The additive used is vinyl carbonate.

##### Separator

It is a thin porous material that physically separates the two electrodes. In this study, polypropylene was used as a separator.

#### Non-cell components

The non-cell components comprise of the BMS, packaging and the cooling system. The data of BMS, packaging and cooling system in this work are procured from Ellingsen et al.^68^

BMS is responsible for the electrical components of a battery system like- the state of charge (SOC), state of health (SOH), temperature, etc. These electrical components ensure safety of the battery system and the electric vehicle on the whole. According to Ellingsen et al.,^68^ the packaging has three categories- module packaging, battery retention and battery tray. The cooling system, as the name suggests, helps in the thermal management of the battery system with an aluminium radiator constituting the primary parts of the system.

#### Cell production

The LIB production includes the following steps, Figure 8:

• The production of the anode and cathode paste requires the mixing of active materials (anode and cathode, separately) with binders PVDF and additives (carbon black) for better conductivity in a solvent NMP (N-Methyl-2-pyrrolidone).
• These electrode pastes are coated to the metal foils and dried (the evaporated NMP solvents are recovered). Copper foil is used for the negative electrode (anode) and aluminium foil is used for the positive electrode (cathode).
• The dried anode and cathode paste on the metal foils are calendered. It is a process where the electrode pastes layers on the respective metal foils are compressed between two moving heated rolls. This technique enables the cast electrodes smoothen the electrode pastes to a required thickness and porosity.
• It is followed by slitting where the foils are cut into the size of the electrodes to make it fit into the prismatic cell. Finally, to remove all the solvents the cut-electrodes are dried again before proceeding to the next step, cell assembly.
• For the prismatic cell, the electrodes are placed on top of each other (stacking) repeatedly. During stacking, a separator, polyolefin, is placed between the anode and the cathode until a prismatic cell of a standard dimension is formed.
• These sandwiched layers of the anodes, separators and the cathodes are then ready for welding. The strips of tabs (Cu tab for anode and Al tab for cathode) connects the electrodes to their respective terminals. The anodes and the Cu tabs are welded together to the negative terminal. The Al tabs are welded to the cathodes and are connected to the positive terminal. Each tab connects to the module’s busbar.
• Once the electrodes are stacked, they are housed in a prismatic enclosing. It is then followed by filling of the cell with an electrolyte, lithium hexafluorophosphate (LiPF_6_), in an established partial vacuum created in the cell. This step ensures proper wetting in vacuum conditions. After the electrolyte is filled, the prismatic cell is sealed under vacuum and is ready for the final stage of cell production, cell finishing.
• The cell formation step includes the first charging and discharging processes in the final stage of cell production. It is one of the most important steps of cell fabrication where the solid electrolyte interphase (SEI) layer is formed through this process.

#### Electricity production mix of the Visegrad countries and Norway

In this work, the electricity was modelled according to the data sourced from Energy-Charts^70^ and ecoinvent (v3.10).^69^ The electricity modelled datasets were subsequently integrated into the LCA study of the NMC 811 battery production. The country’s average electricity mix was used for modelling the electricity mix in SimaPro. Following are the detailed steps for electricity modelling for each country.

##### Data procurement of electricity mix of each country

The electricity production for each country from 2019 to 2024 was sourced from Energy-Charts website, and matched with the electricity production from ecoinvent production processes. For example, electricity production from nuclear source in the Czech Republic will be “Electricity, high voltage {CZ}| electricity production, nuclear, pressure water reactor | Cut-off, U” in ecoinvent.

##### Normalisation, electricity share calculation and integration in SimaPro using ecoinvent database

The total electricity production data from Energy-Charts given in TWh was normalised to 1 kWh and each source contributes proportionately as it is distributed in the Energy-Charts website and in the ecoinvent database. Some electricity production sources were clubbed together or allocated separately to the closest probable choice for better consistency and unification. For example, waste renewable from Energy-Charts was clubbed in Treatment of Blast Furnace in ecoinvent.

##### Electricity imports inclusion

Imports of electricity from other countries were included according to the proportion with respect to the total electricity production.

##### Voltage transformation and losses

The high voltage process then goes through electricity transformation to medium voltage and the numbers are adjusted accordingly with losses during transformation considered separately (ecoinvent).

Among all the other countries represented here, Poland has the highest contribution from coal amounting to 71% of its total production in 2024. Another notable point to be mentioned is, Slovakia ranks first in the chart when it comes to electricity production from nuclear source of energy, followed by Hungary and the Czech Republic. Norway sets an example as being the country with the most share of electricity production generated from the renewable sources of energy, 99%. It is for the same reason this is set as the baseline for setting up a Gigafactory in Norway. Table 4 is based on the data from Energy-Charts 2024 electricity production.^70^ It displays the share of electricity mixes (excluding imports) from the Czech Republic (CZ), Hungary (HU), Poland (PL), Slovakia (SK) and Norway (NO).

**Table 4:** Electricity production mix (TWh) of the Visegrad countries and Norway in 2024, without electricity import (energy charts, 2024)

	CZ	HU	PL	SK	NO
Nuclear	28.04	15.14	0	18.19	0
Hydro run-of-river	1.03	0.1	1.53	4.34	30.19
Hydro water reservoir	1.53	0.10	0.38	0.39	106.18
Wind onshore	0.67	0.63	23.48	0	14.53
Solar	3.91	5.69	17.34	0.54	0
Waste renewable	0.1	0.07	0	0	0.15
Other renewables	2.39	0.12	0	0.47	0.40
Biomass	2.22	0.92	2.36	0.89	0
Fossil brown coal/lignite	23.05	2.35	32.90	0.06	0
Fossil coal-derived gas	0.10	0	1.05	0	0
Fossil hard coal	0.64	0.26	56.15	0.19	0
Fossil gas	3.39	6.40	16.51	2.44	1.63
Fossil oil	0	0	2.04	0.36	0
Waste non-renewable	0.10	0.07	0	0	0.15
Others	0.61	0.44	3.26	0.95	0
Total	67.80	30.907	157.11	28.82	153.24

CZ, Czech Republic; HU, Hungary; PL, Poland; SK, Slovakia; NO, Norway.

Table 4 shows the breakdown of the electricity generation sources- nuclear, hydropower, wind, solar, coal, oil, gas and waste (without considering electricity imports). In the case of the Czech Republic, it has the highest contribution coming from coal (brown coal and hard coal) with a fair share powered by nuclear energy. Figures 3 and 4 complements Table 4 as it gives a better understanding of the sources clubbed as renewable, nuclear and fossil. In terms of the amount of electricity produced from nuclear sources, the Czech Republic produced 28.04 TWh of electricity sourced from nuclear energy. It is followed by Slovakia, 18.19 TWh, and Hungary, 15.14 TWh. However, with 63% of its total share coming from nuclear energy, Slovakia tops the chart among the Visegrad countries. Hungary and the Czech Republic stands at 47% and 41%, respectively.

As mentioned previously, the bar charts illustrating the electricity production mix from 2015-2024 (Visegrad countries and Norway) are depicted in Figures 3 and 4. The electricity sources were clubbed together as renewable, nuclear and fossil. The bar charts depict the changes made in the electricity mix share over the period of ten years primarily to see notable shifts in their electricity production, particularly a transition from fossil-based source to renewable energy sources. Taking each country individually, the Czech Republic maintained a similar pattern in the three categories in terms of percentage share. However, the reliance on coal powered plant has reduced from 54.31% in 2015 to 41.13 in 2024. Not a significant change was seen in the category of renewable and nuclear source of electricity production. Hungary, on the other hand, invested on the renewable share. In 2024 it stood at 23.61%, a big jump from 6.38% in 2015. There has been a gradual increase in this category of electricity production. The nuclear source was 53.68% in 2015 and it is now down to 46.84% in 2024. The dependence on fossil-based energy has decreased to 29.55% from 39.94% in the last ten years. As stated earlier, Poland is heavily reliant on fossil-based sources for its electricity production. Nevertheless, it should also be highlighted that the dependency on fossil-based source, especially coal, has reduced significantly from 94.61% in 2015 to 71.23% in 2024. Another notable thing about the source of electricity production is coming from the renewable source, from 9.39% in 2015 the share is now 28.77%, a big jump and the most, in terms of percentage, among the Visegrad countries. Poland has no nuclear source for electricity production. The electricity mix of Slovakia has the least fossil-based source, 13.85% among all these four countries in 2024. Together with the renewable and nuclear sources (23.01% and 63.14%), they form 86.15% in 2024. The grid profile from 2015-2024 shows a gradual improvement in all the three categories. Norway, on the other hand, has electricity mix with 99% coming from renewable sources, mainly from hydro-, and 1% from fossil-based sources.

### Quantification and statistical analysis

No statistical methods, sample sizes, and software was used generated in the study.

## Supplemental information

Document S1. Figures S1 and S2

Table S1. NMC 811 battery inventory (A) Battery specs. (B) NMC 811_Pack_Cell. (C) Packaging. (D) BMS. (E) Cooling system.
