# Supplementary material for: Regional variations in environmental impacts of NMC 811 production: Comparative LCA study across Visegrad countries
Source: iScience. 2026 Feb 20;29(3):115110. doi: 10.1016/j.isci.2026.115110 (PMC12992527; doi:10.1016/j.isci.2026.115110)
Supplement: Document S1. Figures S1 and S2 [file mmc1.pdf]

iScience, Volume *29*

Supplemental information

Regional variations in environmental impacts of NMC 811 production: Comparative LCA study across Visegrad countries

Thaiskang Jamatia, Viera Pechancová, Debashri Paul, Jens Buchgeister, Manuel Baumann, Hüseyin Ersoy, Merve Erakca, and Petr Saha

Supplemetary: Document S1. Figure S1-S2

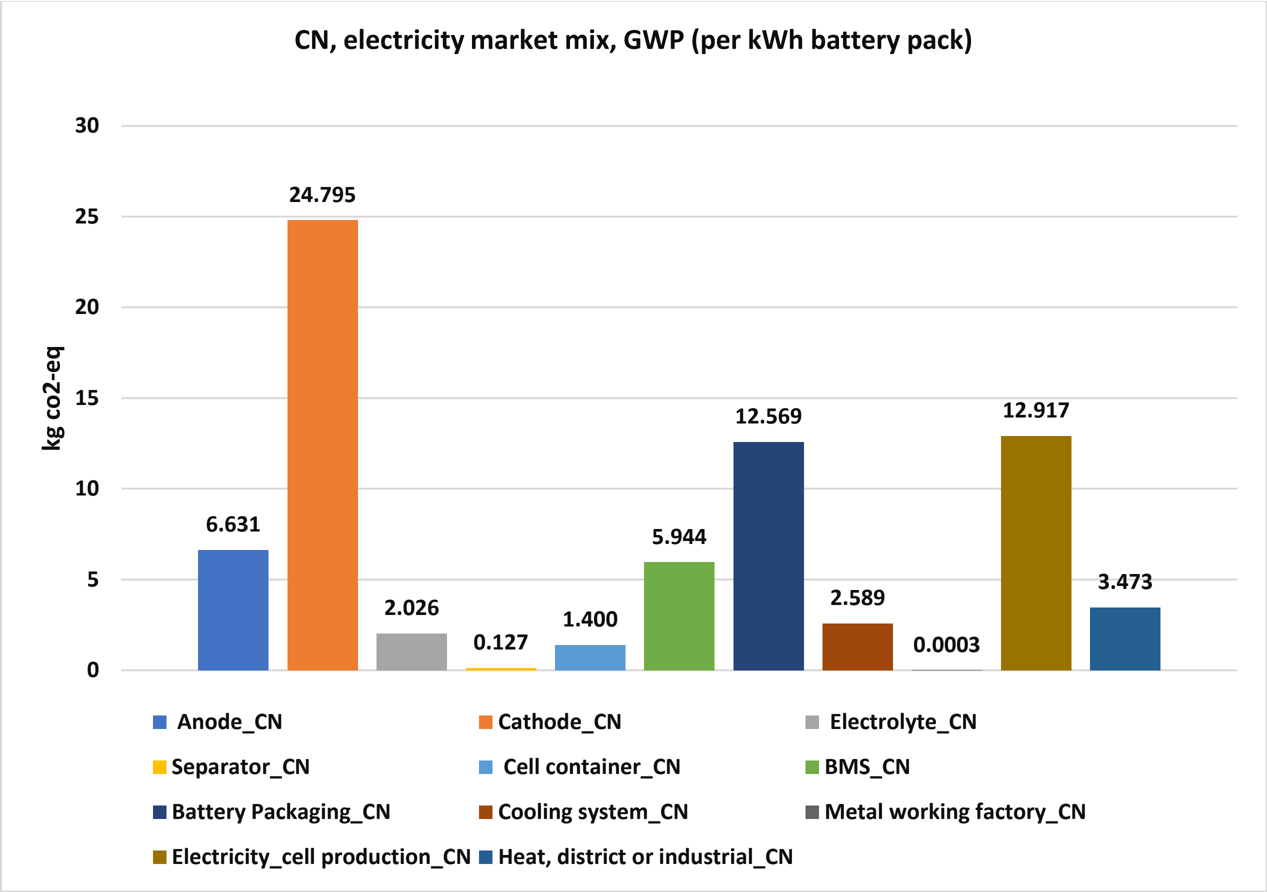

Figure S1. GWP values of NMC 811 battery pack production with China electricity market mix.

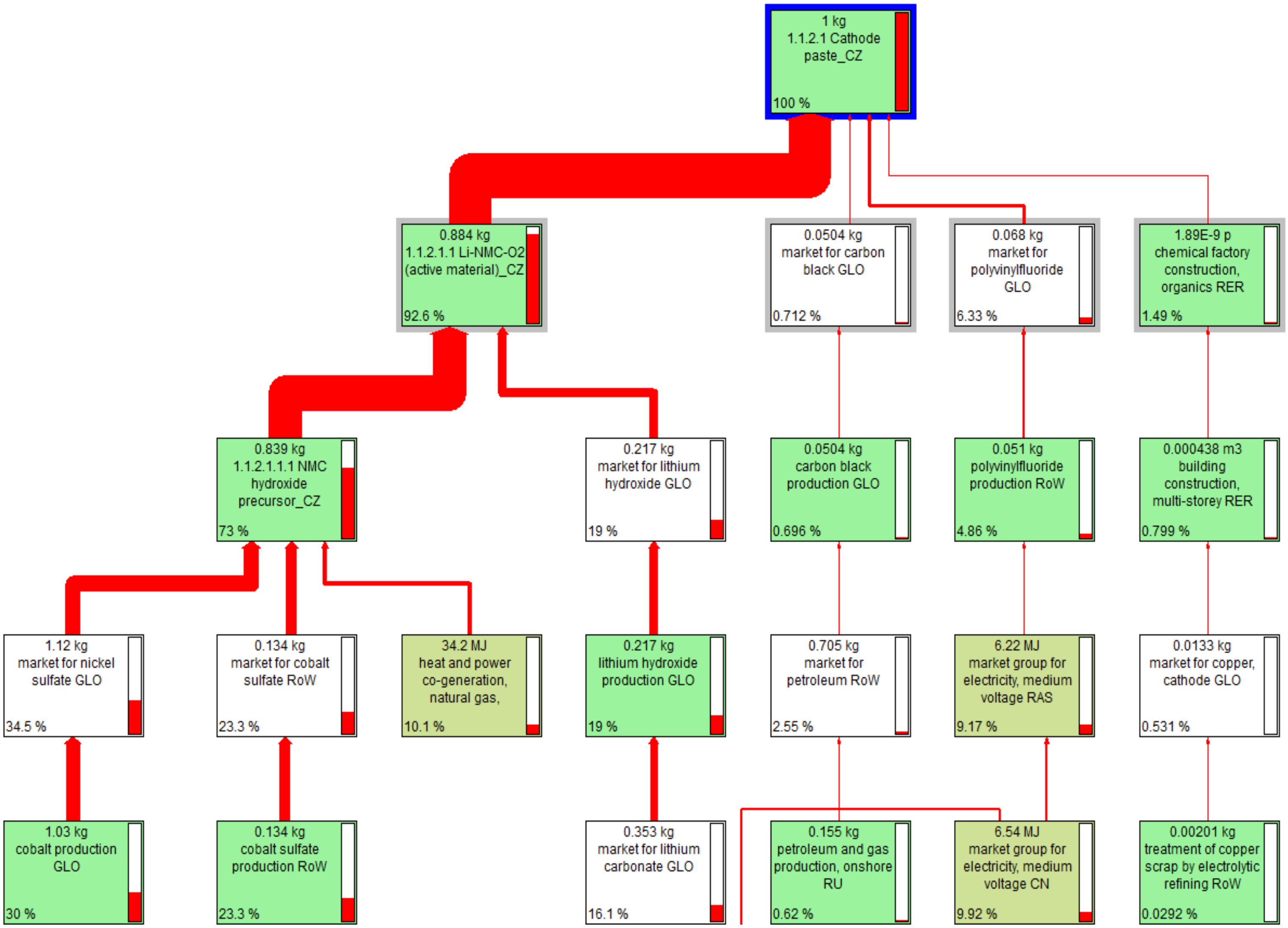

Figure S2. GWP share for cathode active material production.
